# Study on the Extrusion Molding Process of Polylactic Acid Micro Tubes for Biodegradable Vascular Stents

**DOI:** 10.3390/polym14224790

**Published:** 2022-11-08

**Authors:** Yunbo Wei, Jiangeng Bai, Haitao Zhao, Rui Wang, Hongxia Li

**Affiliations:** 1 Department of Mechanical Engineering, Liaoning University of Technology, Jinzhou 121001, China; 2 Department of Mechanical Engineering, Dalian University of Technology, Dalian 116024, China

**Keywords:** polylactic acid, microtube extrusion, extrusion die, extrusion process, biodegradable polymeric stent

## Abstract

Polylactic acid (PLA) has been widely used in the field of medical devices. However, few studies have been conducted on the extrusion molding of PLA micro tubes for the preparation of biodegradable vascular stents. In this paper, the extrusion die for PLA single-cavity micro tubes was designed and manufactured by micro-extrusion theory. Taking the outer diameter, wall thickness, wall thickness uniformity and ovality of micro tubes as the evaluation index, the influence of the main extrusion process parameters on the evaluation index was studied. The experimental results show that the outer diameter and wall thickness are significantly affected by screw speed, pulling speed and gas flow rate; extrusion process parameters have little influence on wall thickness uniformity and ovality within a certain range, which mainly depends on the processing accuracy and assembly accuracy of the extrusion die. However, excessively high screw speed and low gas flow rate have significant effects on ovality. Finally, according to the influence of extrusion process parameters on the evaluation index, a series of micro tubes that meet the design requirements are extruded and carved into vascular stent structures.

## 1. Introduction

Biodegradable polymer stents are one of the most promising directions in the field of interventional medical devices for the future, which are mainly manufactured by the laser machining of thin-walled single-cavity micro tubes. Polylactic acid (PLA) is gradually used as the main material in biodegradable polymer vascular stents due to its good biocompatibility, biodegradability and renewability [1,2,3]. In addition, PLA has the characteristics of light weight, large deformation, strong plasticity and low manufacturing cost, showing broad application prospects in medical and health, electronic communications, aerospace and other fields [4,5,6]. W. Yang et al. [7,8,9,10,11] blended PLA with other materials, such as lignin nanoparticles, nanocrystalline cellulose, chitin nanoparticles, and organically modified montmorillonite powder, etc. to improve its toughness and biodegradability. Malwela et al. [12] studied the degradation rate of PLA with different components. Pooja Bhati et al. [13] prepared porous PLA tubes by extrusion foaming, and studied the effects of different extrusion process parameters on the morphological characteristics and surface hydrophilicity of porous PLA tubes. It can be seen that although some progress has been made in the research of PLA, it is mainly focused on improving its thermodynamic properties, degradation properties and hydrophilic properties. There are few studies on the extrusion of PLA micro tubes.

In recent years, polymer microtubule extrusion molding technology has received more and more attention with the rapid development of minimally invasive interventional medical technology and the increase in demand for medical interventional catheters. However, due to the small size of the micro tubes, it is different from the traditional extrusion molding in terms of mold design, mold manufacturing and extrusion process. In particular, the medical micro tube has higher requirements for geometric accuracy, which puts forward higher requirements for micro tube extrusion molding. Therefore, polymer micro tube extrusion technology has become the focus of scholars’ research. Jin et al. [14,15] separately designed and manufactured single-lumen and multi-lumen micro extrusion die. Polypropylene (PP) or thermoplastic polyurethanes (TPU) was taken as a material, and the effects of extrusion process parameters on shape accuracy were studied. Tian et al. [16] focused on the extrusion process of polymer multi-lumen micro tubes and established a pre-compensation design method of micro tubes extrusion die based on the parison deformation control. PP was taken as a material, and the feasibility and effectiveness of the proposed method were validated through extrusion process experiments of micro tubes. According to the above-mentioned studies, most of the existing research has taken PP, TPU and other hose materials with relatively good fluidity as the research object. However, relatively few papers are devoted to the studies on the extrusion process of PLA micro tubes, and little is known about the effects of extrusion process parameters on PLA microtubule dimension and shape precision.

In this paper, PLA micro tubes were taken as the research object. Firstly, the rheological properties of PLA were studied, and extrusion die for single-cavity micro tubes was designed and manufactured through micro-extrusion theory. The influence of extrusion process parameters on PLA microtubule dimension and shape precision and its mechanism were studied through experiments of the extrusion process. Finally, by adjusting the extrusion process, a series of micro tubes with different dimensions that meet the design requirements were extruded and carved into vascular stent structure.

## 2. PLA Capillary Rheology Experiments

The rheological properties of polymer materials are the basis of polymer molding, which is of great significance to the design of extrusion die and the research of extrusion process. Capillary rheological experiment is an important method to determine the rheological properties of materials. Therefore, the capillary rheological experiment of PLA was carried out firstly.

### 2.1. Experimental Equipment and Methods

A twin bore capillary rheometer (Rosand-RH7D) made by Malvern instruments Ltd. (Malvern, UK) was used for the experiments. Long length die with diameter of 0.5 mm and corresponding zero length die were selected. PLA (NatureWorks 4032D) was dried in drying box for 4 h at 79 °C before the test, then the rheological properties of the PLA were tested under the temperature of 190 °C, 200 °C, 210 °C, 220 °C, respectively.

### 2.2. Results of the Rheological Experiments

**Figure 1:**
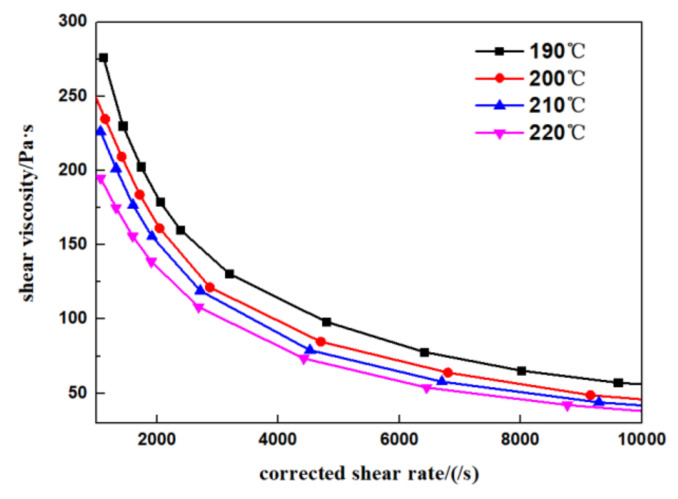
Rheological characteristic curves of PLA.

The experimental results are shown in Figure 1. Shear viscosity of PLA decreases with the shear rate, which means PLA is a pseudoplastic fluid. When the shear rate is less than 8000 s^−1^, shear viscosity decreases exponentially with the increase of shear rate. However, with the further increase of shear rate, the downward trend tends to be gentle. Shear viscosity decreases with the increase of temperature, but when the temperature is higher than 200 °C, the difference in shear viscosity at different temperatures is very small.

## 3. Design and Manufacture of Extrusion Die for PLA Micro Tubes

**Figure 2:**
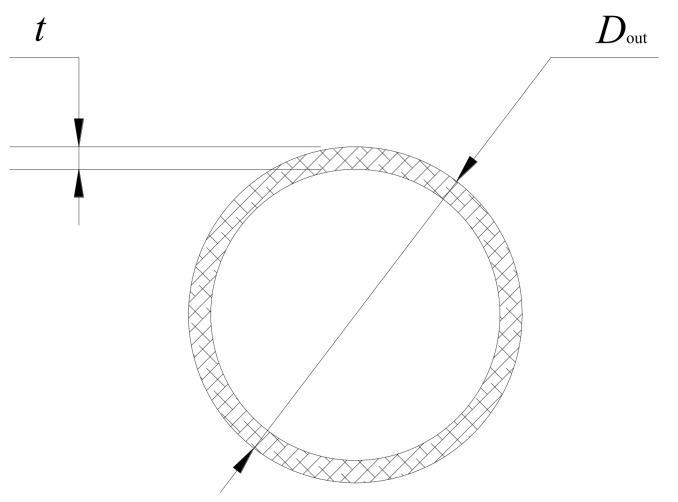
Section of the PLA micro tube.

The section of designed PLA micro tubes is shown in Figure 2. The outer diameter *D*_out_ is within the range of 1.00~2.50 mm, wall thickness is within the range of 0.05~0.15 mm, Wall thickness uniformity > 90% and ovality < 5.0% were required for the micro tubes.

### 3.1. Structure of the Extrusion Die

**Figure 3:**
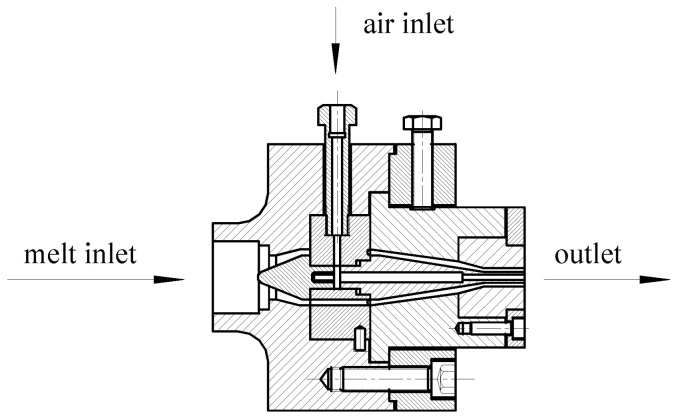
The in-line extrusion die structure for micro tubes.

An in-line extrusion structure was adopted for extrusion die. A micro gas injection system was introduced because of the tiny size of the designed micro tubes, and the gas injection direction is perpendicular to the melt flow direction [14]. The structure of the extrusion die is shown in Figure 3.

### 3.2. Calculation of Inner Diameter of Die Land and Outer Diameter of Mandrel Land

The inner diameter of the die land and the outer diameter of mandrel land are critical to the dimension of the extruded micro tubes. It will be impossible to achieve the required tube dimension by adjusting the extrusion process if the two parameters are too large or too small. Nevertheless, there is no accurate formula for the calculation of the two parameters for micro-scale extrusion die. Usually, they are determined by experience, combined with the draw ratio of material. The draw ratio can be calculated according to Equation (1). The draw ratio of PLA ranges from 1.1 to 4.8, which is determined through the previous extrusion process experiments. The inner diameter of the die land and outer diameter of mandrel land were initially determined to be 3.40 mm and 3.00 mm, respectively, according to the experiment. Then, the required maximum and minimum draw ratios which satisfy the requirements of the designed microtubule dimensions can be obtained by calculation. The calculated maximum draw ratio was less than 3.44, and the minimum draw ratio was greater than 1.36, which were within the allowable range of the material.

(1) $$I=\frac{D_{1}+d_{1}}{D_{\mathrm{out}}+d_{in}}$$

where I is draw ratio, $D_{1}$ and $d_{1}$ are respectively outer diameter of the die land and inner diameter of the mandrel land, *D*_out_ and *d*_in_ are respectively outer diameter and inner diameter of the designed micro tubes.

### 3.3. Calculation of Land Length

Land length has a significant effect on the quality and production efficiency of the micro tubes. If the land length is too short, the extrudate swell will be serious, and the micro tubes cannot be extruded. On the contrary, if the land length is too long, productivity will be reduced, because of too much pressure loss, and the melt will be easily decomposed or degraded due to the excessive residence time of the melt in the extrusion die.

**Figure 4:**
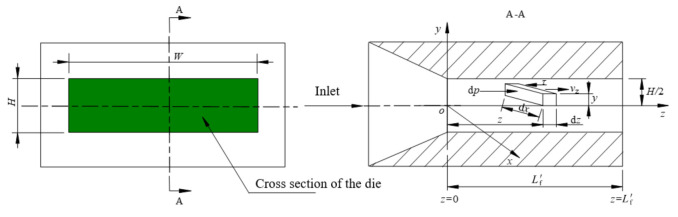
The flow channel structure of slit-shaped die.

The flow channel structure of slit-shaped die is shown in Figure 4. It is assumed that there is a fluid unit at a certain distance *z* from the entrance of the die *z* = 0, which flows towards the exit of the die under the action of pressure $d_{p}$, and is also affected by the shear stress between the polymer fluid layers. According to the principle of force balance, it can be obtained that [17]:

(2) $$y\cdot d_{x}\cdot d_{p}=d_{z}\cdot d_{x}\cdot \tau$$

Equation (2) can be converted to

(3) $$\tau =y\cdot \frac{d_{p}}{d_{z}}$$

For pseudoplastic polymer fluids, the power-law function equation is:

(4) $$\tau =-K\cdot {\left(\frac{d_{v_{z}}}{d_{y}}\right)}^{n}$$

where τ is shear stress, K is fluid consistency and n is non-Newtonian index.

Combined with the Equations (3) and (4), the volume flow rate of the polymer melt in the slit-shaped die is finally obtained:

(5) $$Q_{v}=\frac{2n}{2n+1}{\left(\frac{\Delta P}{KL}\right)}^{\frac{1}{n}}\cdot {\left(\frac{H}{2}\right)}^{\frac{2n+1}{n}}\cdot W$$

where $Q_{v}$ is the volume flow rate, ΔP is the pressure drop, L is the length of the forming section, K is the fluid consistency, n is the non-Newtonian index, W is the slit width, and H is the slit thickness.

The length of the die can be derived from Equation (5):

(6) $$L=\frac{H\Delta P}{2K{\left(\frac{4n+2}{n}\right)}^{n}\cdot {\left(\frac{Q_{v}}{WH^{2}}\right)}^{n}}$$

In general, the die can be considered as a slit-shaped die when W/H ≥ 10 is satisfied.

For the designed microtubule extrusion die, H is the gap between the die and the mandrel, and W is the average circumference of the ring formed by the die and the mandrel, according to Equations (7) and (8), H = 0.2 mm, W = 10.05 mm, W/H = 50.25 > 10, therefore, the land length can be calculated on the basis of the theory of slit-shaped die.

(7) $$H=D_{1}-d_{1}$$

(8) $$W=\pi \times \left[\frac{D_{1}}{2}+\frac{d_{1}}{2}\right]$$

where $D_{1}$ and $d_{1}$ are respectively the inner diameter of die land and outer diameter of mandrel land.

K and n in Equations (5) and (6) were obtained by extracting the rheological data of PLA at a certain temperature and fitting the power law equation of non-Newtonian fluid (Equation (9)). ΔP and $Q_{v}$ were obtained through experiments. After calculation, n = 0.306, K = 23.79 kPa, ΔP = 14.3 Mpa, $Q_{v}$ = 129.6 ${\mathrm{mm}}^{3}/s$.

(9) $$\tau =K{\dot{\gamma }}^{n}$$

where τ is shear stress, K is fluid consistency, $\dot{\gamma }$ is shear rate, n is non-Newtonian index.

Finally the land length L ≈ 5.00 mm was achieved by substituting W, H, n, K, ΔP, $Q_{v}$ into Equation (6).

### 3.4. The Manufacture of Extrusion Die

**Figure 5:**
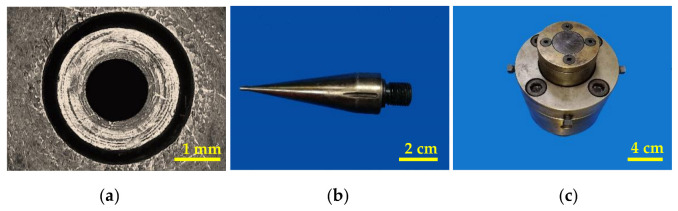
The extrusion die for PLA micro tubes: (**a**) The cross section of extrusion die; (**b**) Mandrel; (**c**) Assembly of extrusion die.

The difficulties of machining extrusion die are mainly in the processing of mandrel forming land and vent holes in the mandrel. Machining of outer surface of the mandrel was carried out by turning-milling machining center, and he machining of the gas injection hole was conducted by wire cutting processing. The extrusion die is shown in Figure 5.

## 4. Extrusion Process Experiments of PLA Micro Tubes

### 4.1. Experimental Equipment and Methods

The extrusion equipment was single extruder system (Model: HPE-100H, Davis-Standard LLC, Pawcatuck, CT, USA). The dimension measuring device was the digital-image-tool microscope (Model: VTM-3020F, Suzhou Aoka Optical Instrument Co., Ltd., Suzhou, China) with the measurement accuracy of ±0.001 mm. The gas flow control device was the gas mass flow controller (Model: FLC-100D, Shenzhen Flow Method Measure & Control Systems Co., Ltd., Shenzhen, China), unit: g/min.

PLA (NatureWorks 4032D) was dried in a drying box for 4 h at 79 °C before the tests. The barrel temperature from the inlet to the outlet of the extruder were set respectively at 180 °C, 190 °C, 195 °C on the basis of the properties of PLA and the plasticizing performance of the extruder, and kept the barrel temperature unchanged during the experiments. The effects of screw speed, pulling speed, die temperature, gas flow rate and vacuum degree on the outer diameter, wall thickness, wall thickness uniformity and ovality of micro tubes were studied by single factor method. Sixteen points were selected evenly from the cross sections of the micro tubes in the circumferential direction, then the outer diameter and wall thickness at each selected point were measured by tool microscope. The measured average values were considered as the dimensions of the micro tube outer diameter and wall thickness. Wall thickness uniformity can be calculated according to Equations (10)–(12), ovality can be calculated according to Equation (13) [18].

(10) $$G=100\%-\frac{S_{\mathrm{td}}}{\bar{\delta }}\cdot 100\%$$

(11) $$S_{\mathrm{td}}=\sqrt{\frac{1}{n-1}\sum_{i=1}^{n}(\delta_{i}-\bar{\delta }{)}^{2}}$$

(12) $$\bar{\delta }=\frac{1}{n}(\delta_{1}+\delta_{2}+\ldots +\delta_{n})$$

where G is wall thickness uniformity, $S_{\mathrm{td}}$ is standard deviation of wall thickness, $\delta_{i}$ is wall thickness measured at each point in the circumferential direction of the micro tubes, $\bar{\delta }$ is the average value of the wall thickness, *n* is the number of the measured point.

(13) $$Y_{0}=2(D_{\max}-D_{\min})/(D_{\max}+D_{\min})$$

where $Y_{0}$ is ovality, $D_{\max}$ and $D_{\min}$ represent the maximum and minimum outer diameter of the micro tubes, respectively.

To prevent the failure of the experiment due to unreasonable parameters, such as melt degradation, microtubule tensile fracture or rupture, the range of the extrusion process parameters were determined through rheological experiments and preliminary exploratory experiments, which are shown in Table 1.

### 4.2. Results and Discussion

(1) Effects of screw speed on evaluation index of PLA micro tubes.

**Figure 6:**
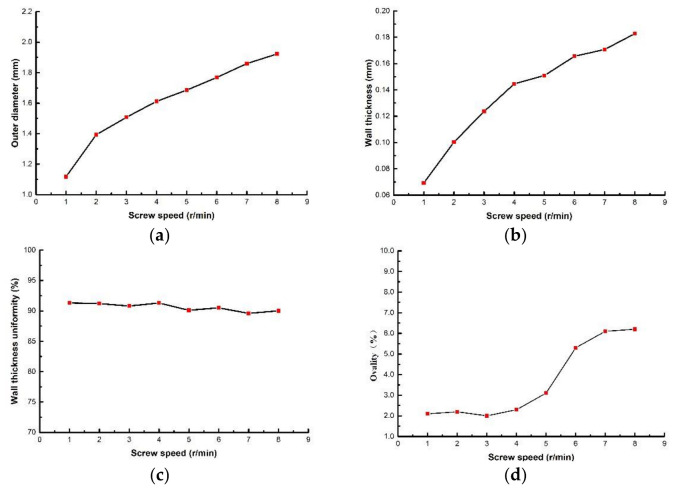
The relationships between evaluation index with screw speed: (**a**) The relationship between outer diameter with screw speed; (**b**) The relationship between wall thickness with screw speed; (**c**) The relationship wall thickness uniformity with screw speed; (**d**) The relationship between ovality with screw speed.

**Figure 7:**
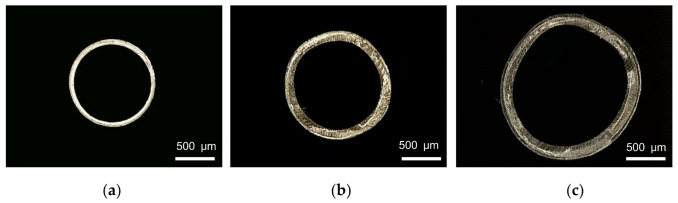
Cross section of micro tubes at different screw speeds: (**a**) Screw speed = 1 r/min; (**b**) Screw speed = 5 r/min; (**c**) Screw speed = 8 r/min.

The screw speed has significant effects on the melt volume flow rate, since the melt is extruded from extruder through screw rotation. When studying the influence of screw speed on the evaluation index, the rotational speed of the extruder ranges from 1 to 8 r/min, and the die temperature, pulling speed, gas flow rate and vacuum degree are 200 °C, 10 m/min, 22 g/min and 0 inH_2_O, respectively. The relationship between the screw speed and the evaluation index is shown in Figure 6. It can be seen that both outer diameter and wall thickness of the micro tubes increase nonlinearly, and the increase trend become gradually slower with the increase of screw speed. When the rotational speed is lower than 4 r/min, the extruder rotational speed has little influence on the wall thickness uniformity and ovality. With the continuous increase of the extruder rotational speed, wall thickness uniformity decreases slightly and ovality increases significantly. Figure 7 shows the cross-section of micro tubes extruded at the screw speed of 1 r/min, 5 r/min and 8 r/min, respectively.

There are two main reasons for the increase of microtubule outer diameter and wall thickness. On one hand, the increase of the screw speed leads to the increase of the melt volume flow rate, then the shear rate increases. For a pseudoplastic fluid, extrusion swelling ratio usually increases with the increase of the shear rate before melt fracture in the extrusion process [19]. Therefore, outer diameter and wall thickness increase with the increase of the screw speed. On the other hand, according to the continuity theorem:

(14) $$V_{0}\times S_{0}=V\times S$$

where $V_{0}$ and $S_{0}$ are separately melt flow rate in the cavity and sectional area of the cavity. *V* and *S* are separately pulling speed and sectional area of final micro tubes. An increase in screw speed means an increase in $V_{0}$, while $S_{0}$ and *V* do not change, then *S* increases, that is, the wall thickness increases. Since the inner wall of the micro tubes cannot move inward under the gas injection pressure, the outer diameter of the micro tubes also increases. With the increase of screw speed, the shear rate of the melt increases resulting in shear thinning, which makes the increase of the melt volume flow rate slow down. Therefore, the increase in micro tubes outer diameter and wall thickness becomes gradually slower. In order to verify this point of view, the capillary rheological experiments were carried out.

The volume flow rate of capillary die can be expressed as

(15) $$Q_{v}=(\frac{n\pi }{3n+1})\dot{\gamma }R^{3}$$

where $Q_{v}$ is volume flow rate, *n* is non-newton index, $\dot{\gamma }$ is shear rate, *R* is radius of capillary die.

Capillary die is radius *R* is 0.25 mm. According to rheological data at any temperature by rheological experiments, the volume flow rate can be obtained by extracting the *n* and $\dot{\gamma }$ at different compression rates and substituting them into the formula.

**Figure 8:**
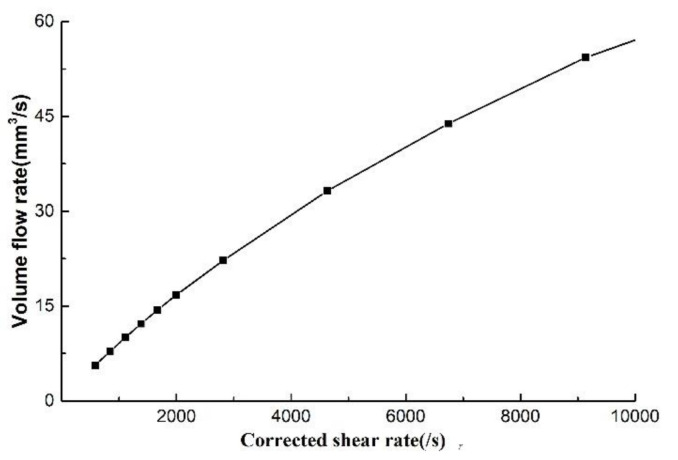
The relationship between shear rate and volume flow rate.

For example, when the melt temperature is 200 °C, rheological experimental data are extracted to obtain *n*, $\dot{\gamma }$ and calculated shear rates at different compression rates, as is shown in Table 2. The relationship between shear rate and volume flow rate is shown in Figure 8. It can be seen that the volume flow rate increases with the increase of shear rate, and the increasing trend gradually slows down, which verifies the correctness of the proposed viewpoint, a the viewpoint is consistent with the extrusion molding of PP micro tubes [20].

It is assumed that the gap between the die and the mandrel is equal at the extrusion die forming section, and the melt flow is balanced so each point on any concentric circle of the cavity cross section has the same velocity field and stress field. In theory, the wall thickness uniformity and ellipticity of the micro tubes are not affected as long as the gap between the die and the mandrel in the forming section is equal. Therefore, the shape accuracy of the micro tube mainly depends on the processing accuracy and assembly accuracy of extrusion die. However, in the actual extrusion process, there must be a certain error between the die and mandrel in the processing and assembly, which results in a gap in the molding section is not exactly equal, and with it the rotational speed of the extruder, the shear rate of the melt, the expansion rate, the wall thickness uniform and ovality increase. However, it is not obvious when the screw speed is at a low level. In addition, due to the increase of the screw speed and volume flow rate, the cooling and solidification rate become slow, leading to deformation under the influence of gravity. As a result, the wall thickness uniformity decreases and the ovality increases.

(2) Effects of pulling speed on evaluation index of PLA micro tubes

**Figure 9:**
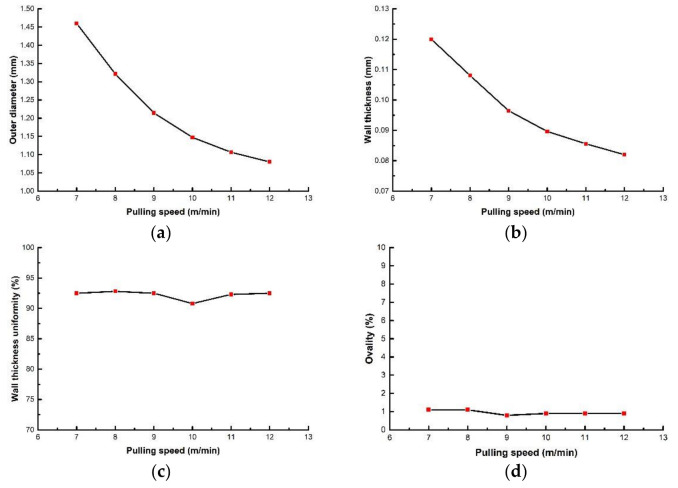
The relationships between evaluation index with pulling speed: (**a**) The relationship between outer diameter with pulling speed; (**b**) The relationship between wall thickness with pulling speed; (**c**) The relationship wall thickness uniformity with pulling speed; (**d**) The relationship between ovality with pulling speed.

**Figure 10:**
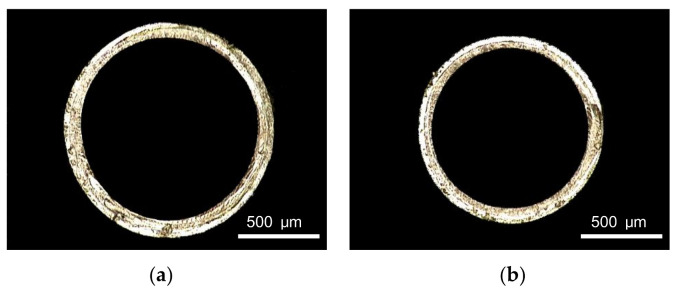
Cross section of micro tubes at different pulling speeds: (**a**) Pulling speed = 7 m/min; (**b**) Pulling speed = 12 m/min.

Micro tubes, under the action of the upper and lower splints of the tractor, make the extruded melt move along the extrusion direction. The pulling speed ranges from 7 m/min to 12 m/min. The screw speed, die temperature, gas flow rate and vacuum degree are 2 r/min, 200 °C, 11 g/min and 0 inH_2_O respectively. The relationships between pulling speed and evaluation index are shown in Figure 9. It can be seen that both the outer diameter and the wall thickness of the micro tubes decrease nonlinearly with the increase of pulling speed. The decrease is significant at the beginning, and with the continuous increase of pulling speed, the decrease trend becomes slower gradually. Pulling speed has little effect on wall thickness uniformity and ovality. Figure 10 shows the cross-section of micro tubes extruded at the pulling speed of 7 m/min, 12 m/min, respectively.

The reduction in the outer diameter and wall thickness of micro tubes can still be analyzed according to Equation (14). As the pulling speed *V* increases, while $V_{0}$ and $S_{0}$ do not change, the *S* inevitably decreases. That is, wall thickness decreases, which leads to an inward contraction of the outer wall, i.e., the outer diameter decreases. Die swell occurs when the melt leaves the die. Deformation caused by die swell is first straightened under the traction force. Therefore, the microtubule outer diameter and wall thickness decrease more obviously at low pulling speed. With the continuous increase of pulling speed, the deformation caused by die swell is gradually weakened, and even disappeared. So, the decrease becomes slow gradually.

The pulling speed mainly affects the tensile ratio of micro tubes, and has little effect on wall thickness uniformity and ovality. However, it is worth noting that the appropriate range of pulling speed should be selected, or the gap of tractor clamping should be adjusted at different pulling speeds when studying the influence of pulling speed on evaluation index. Otherwise, the experiment will fail. The reason is that the micro tubes are large and are easily compressed and deformed by the splint of the tractor at low pulling speed, resulting in an increase of ovality. On the contrary, when pulling speed is too high, the diameter of the micro tubes are too small, leading to the splint can not effectively hold the micro tubes.

(3) Effects of die temperature on evaluation index of PLA micro tubes

**Figure 11:**
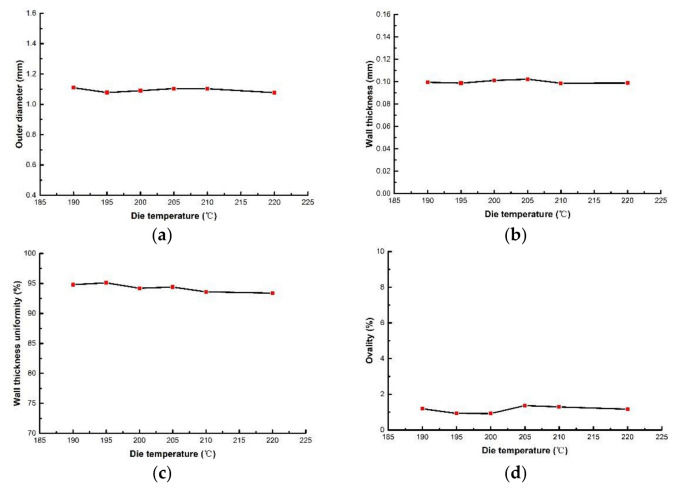
The relationships between evaluation index with die temperature: (**a**) The relationship between outer diameter with die temperature; (**b**) The relationship between wall thickness with die temperature; (**c**) The relationship wall thickness uniformity with die temperature: (**d**) The relationship between ovality with die temperature.

**Figure 12:**
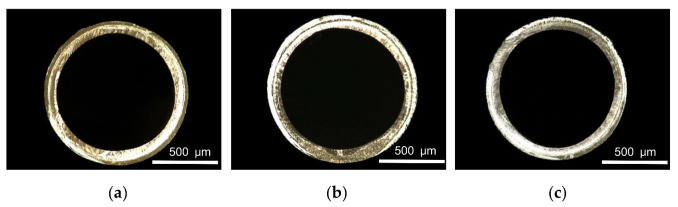
Cross section of micro tubes at different die temperatures: (**a**) Die temperature = 190 °C; (**b**) Die temperature = 205 °C; (**c**) Die temperature = 220 °C.

Die temperature mainly affects the viscosity of the melt. When studying the influence of die temperature on the evaluation index, the die temperature ranges from 190 °C to 220 °C. The screw speed, pulling speed, gas flow rate and vacuum were 2 r/min, 10 m/min, 8 g/min and 0 inH_2_O, respectively. The relationships between the die temperature and the evaluation index are shown in Figure 11. It can be seen that the outer diameter and wall thickness of PLA micro tubes are affected very little by die temperature. When the temperature is lower than 205 °C, the wall thickness uniformity and ovality almost do not change with the increase in temperature. When the die temperature is higher than 205 °C, the wall thickness uniformity decreases slightly, and the ovality increases slightly. Figure 12 shows the cross section of micro tubes at the die temperatures of 190 °C, 205 °C and 220 °C, respectively.

The die temperature mainly affects the viscosity of the melt, however, it has little effect on volume flow rate. Therefore, the outer diameter and wall thickness are minimally affected by temperature. The point of this view can also be verified by rheological experiments.

**Figure 13:**
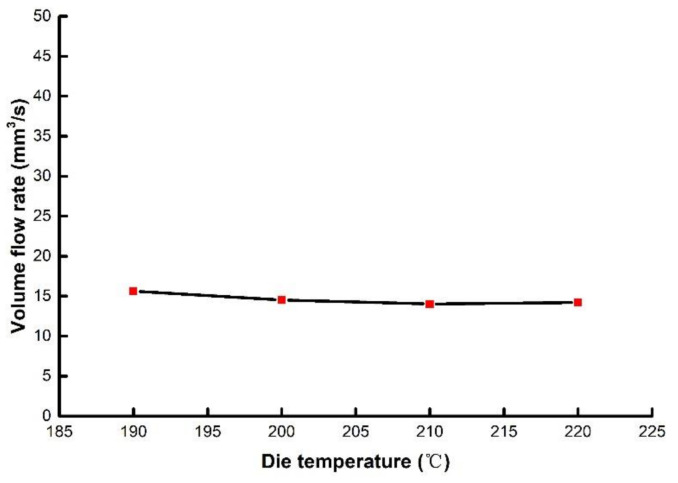
The relationship between die temperature and volume flow rate.

The non-Newton index *n*, corrected shear rate $\dot{\gamma }$ in the rheological data of PLA at temperatures of 190 °C, 200 °C, 210 °C and 220 °C were extracted and substituted into Equation (14). The volume flow rates at different temperatures were obtained by calculation, as shown in Figure 13. It can be seen that as the temperature increases, the melt volume flow rate hardly change. Therefore, the temperature has almost no effect on the outer diameter and wall thickness of micro tubes.

When the die temperature is high, the wall thickness uniformity decreases and the ovality increases slightly. The reason is that when the melt leaves the die, the cooling and solidification rate become slow, leading to deformation under the influence of gravity.

(4) Effects of gas flow rate on evaluation index of PLA micro tubes

**Figure 14:**
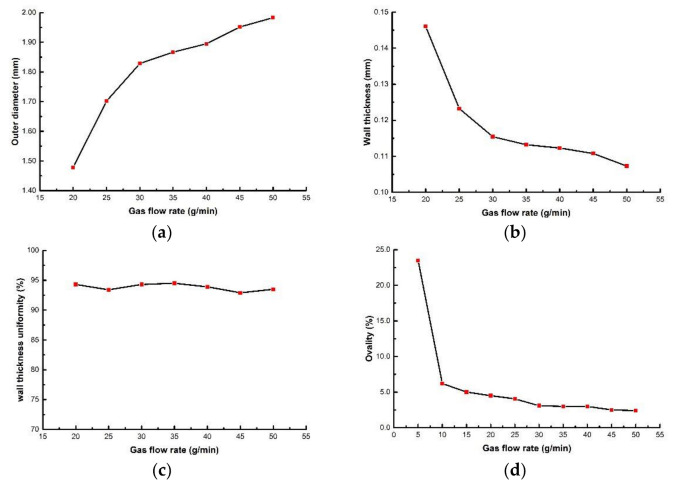
The relationships between evaluation index with gas flow rate: (**a**) The relationship between outer diameter with gas flow rate; (**b**) The relationship between wall thickness with gas flow rate; (**c**) The relationship wall thickness uniformity with gas flow rate; (**d**) The relationship between ovality with gas flow rate.

**Figure 15:**
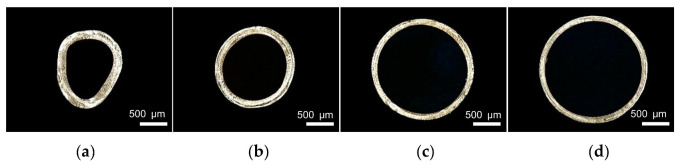
The cross section of micro tubes at different gas flow rates: (**a**) Gas flow rate = 5 g/min; (**b**) Gas flow rate = 20 g/min; (**c**) Gas flow rate = 35 g/min; (**d**) Gas flow rate = 50 g/min.

During the extrusion, gas is injected into the interior of the micro tubes at a certain rate to prevent parison from sticking together for the deformation caused by gravity when the melt leaves the die. When studying the influence of gas flow rate on the evaluation index, the gas flow rate ranges from 5 to 50 g/min. The screw speed, die temperature, pulling speed and vacuum degree were 3 r/min, 200 °C, 8.5 m/min, 0 inH_2_O, respectively. The relationships between gas flow rate and the evaluation index are shown in Figure 14. It is worth noting that when gas flow rate is lower than 20 g/min, billet deformation is serious, so the wall thickness and outer diameter are not measured. It can be seen from Figure 14 that with the increase of gas flow rate, microtubule outer diameter increases and microtubule wall thickness decreases, and both the changes are nonlinear, ahe rate of changes decreases gradually. The effect of gas flow rate on the wall thickness uniformity is small. The ovality decreases with the increase of the gas flow rate. The decrease is significant at the beginning and becomes stable when gas flow rate reaches a certain level. Figure 15 shows the cross section of micro tubes at gas flow rates of 5 g/min, 20 g/min, 35 g/min and 50 g/min respectively.

The increase of the gas flow rate leads to a pressure increase of the parison inner wall, then the parison gradually expands outwardly under the pressure difference of inside and outside parison, meanwhile, the parison becomes thinner. Therefore, the outer diameter of the micro tubes increases and the wall thickness decreases. With the continuous increase of the outer diameter of the micro tubes, the degree of molecular orientation in the circumferential direction of the melt increases, and intermolecular force is enhanced. Therefore, the deformation caused by the gas pressure would be suppressed. As a result, the rate of changes of the outer diameter and wall thickness of the micro tubes gradually decreases with the increase of the gas flow rate.

The effect of gas flow rate on wall thickness uniformity and ovality is mainly reflected in the case of low gas flow rate. When the gas flow rate is at a low level, the gas pressure inside the parison is not sufficient to resist the deformation caused by gravity, which leads to a serious deformation of the micro tubes, therefore the effects of the gas flow rate on wall thickness uniformity and ovality are prominent. When gas flow rate increases to a certain degree, the internal pressure of the parison is sufficient to resist the deformation caused by gravity, then gas flow rate has little effect on wall thickness uniformity and ovality.

(5) Effects of vacuum degree on evaluation index of PLA micro tubes

**Figure 16:**
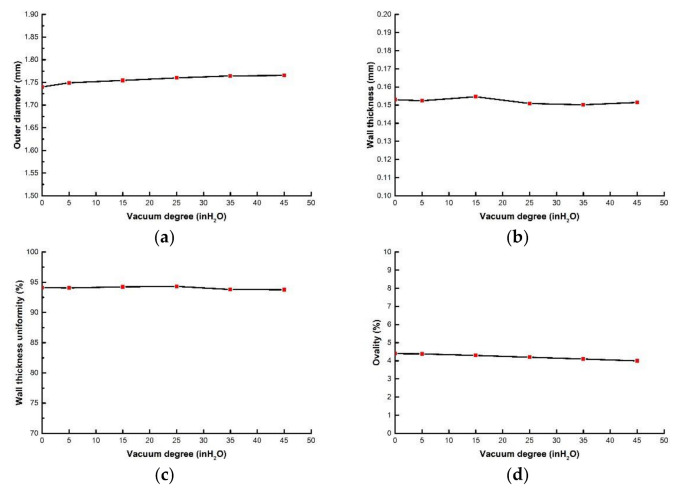
The relationships between evaluation index with vacuum degree: (**a**) The relationship between outer diameter with vacuum degree; (**b**) The relationship between wall thickness with vacuum degree; (**c**) The relationship wall thickness uniformity with vacuum degree; (**d**) The relationship between ovality with vacuum degree.

**Figure 17:**
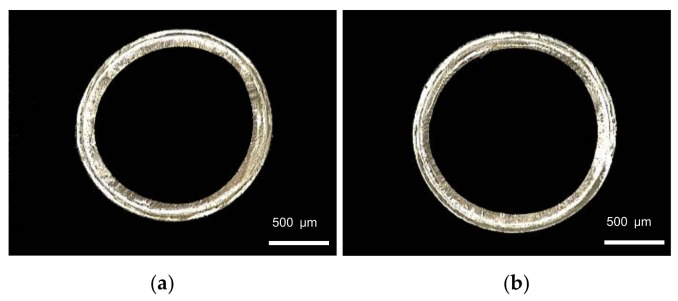
The cross section of micro tubes at different vacuum degrees: (**a**) Vacuum degree = 0 inH_2_O; (**b**) Vacuum degree = 45 inH_2_O.

Vacuum degree refers the pressure of the gas above the water surface in the cooling water tank. When studying the influence of vacuum degree on the evaluation index, the vacuum degree ranges from 0 to 45 inH_2_O. The screw speed, die temperature, pulling speed and gas flow rate were 4 r/min, 200 °C, 9 m/min, 26 g/min, respectively. Figure 16 shows the relationships between evaluation index and vacuum degree. It can be seen that with the increase of vacuum degree, the outer diameter, wall thickness and wall thickness uniformity almost do not change, and the ovality slightly decreases. Figure 17 shows the cross section of micro tubes at vacuum levels of 0 inH_2_O and 45 inH_2_O, respectively.

The vacuum degree mainly affects the pressure in the cooling water. Due to the small size of the micro tubes, the force on the micro tubes is small, so the vacuum degree has little influence on the outer diameter, wall thickness and wall thickness uniformity. With the increase of vacuum degree, the ovality decreases slightly, which is because the micro tubes will have a certain deformation under the influence of buoyancy and water gravity in the cooling water. When the vacuum degree increases, a certain negative pressure will be formed on the cooling water, which will reduce the pressure in the cooling water, thus reducing the deformation of the microtubule products.

**Figure 18:**
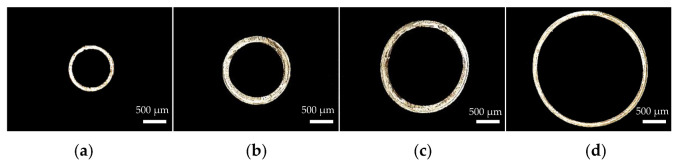
Cross sections of different specifications of micro tubes: (**a**) *D* = 1.00 mm; (**b**) *D* = 1.50 mm; (**c**) *D* = 2.00 mm; (**d**) *D* = 2.50 mm.

**Figure 19:**
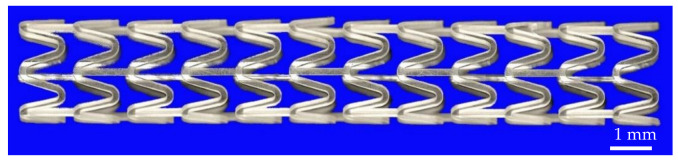
The engraved vascular stent.

In summary, the screw speed, pulling speed and gas flow rate have significant effects on the microtubule outer diameter and wall thickness. The die temperature and the vacuum degree have almost no effects on the microtubule size. Therefore, the required outer diameter and wall thickness of PLA micro tubes can be obtained by coordinating the screw speed, pulling speed and the gas flow rate. The extrusion process parameters have little influence on wall thickness uniformity and ovality within a certain range, which mainly depends on the processing accuracy and assembly accuracy of the extrusion die. However, if the process parameters are too large or too small, wall thickness uniformity and ovality will also be greatly affected. For example, when the screw speed and die temperature are too high and the traction speed is too low, the parison is seriously deformed due to gravity, resulting in poor wall thickness uniformity and large ovality; even the extrusion will be interrupted due to the inability to pull the parison. If the gas flow rate is too small, the deformation caused by the insufficient pressure of the parison inner wall to support gravity will also lead to the reduction of the wall thickness uniformity and the increase of the ovality. If the gas flow rate is too large, the parison will be broken, and the extrusion will be interrupted. With the increase of vacuum degree, ovality decreases, but the variation range is very small. Therefore, in order to obtain better shape accuracy, the screw speed and die temperature should not be too high, the gas flow rate should be sufficient and properly increase the vacuum degree. Accordingly, a series of PLA micro tubes with high shape accuracy that meet the design requirements were processed by adjusting the process parameters. Table 3 lists the specific size and shape accuracy of some representative micro tubes, and the cross-sections are shown in Figure 18. Finally, a micro tube with an outer diameter of 2.50 mm and a wall thickness of 0.15 mm was processed into a vascular stent structure by laser engraving process. During the engraving process, the inability to cut or partially cut did not occur, and the engraved vascular stent is shown in Figure 19.

## 5. Conclusions

In this paper, PLA single-cavity micro tubes were used as the research object. Firstly, the rheological properties of PLA were studied, and extrusion die for single-cavity micro tubes were designed and manufactured through micro-extrusion theory. Taking the outer diameter of single–cavity micro tubes as the evaluation index, the influence of the main extrusion process parameters on the evaluation index was studied. The results show that the main factors affecting the outer diameter and wall thickness of the micro tubes are screw speed, pulling speed. The gas flow rate, die temperature and vacuum degree have almost no effect. Extrusion process parameters have little influence on wall thickness uniformity and ovality within a certain range, which mainly depends on the processing accuracy and assembly accuracy of the extrusion die. However, excessively high screw speed and low gas flow rate have significant effects on ovality, and have little effect on wall thickness uniformity. The pulling speed, die temperature and vacuum degree have little effect on the wall thickness uniformity and ovality. Finally, according to the influence of extrusion process parameters on the size and shape accuracy of microtubes, a series of specifications of PLA single-cavity micro tubes that meet the design requirements were processed, and these micro tubes were successfully carved into vascular stent structures.

## Figures and Tables

**Table 1:** The value ranges of extrusion process parameters.

Extrusion Process Parameter	Value
Screw speed (r/min)	1~8
Pulling speed (m/min)	7~12
Die temperature (°C)	190~220
Gas flow rate (g/min)	5~50
Vacuum degree (inH_2_O)	0~45

**Table 2:** *n*, $\dot{\gamma }$, $Q_{v}$ at different compression rates at 200 °C.

Plunger Compression Rate (mm/min)	Corrected Shear Rate $\dot{\gamma }\;(/s)$	Non-Newton Index *n*	Volume Flow Rate $Q_{v}\;({\mathrm{mm}}^{3}/s)$
2.08	581.21	0.48	5.61
2.92	842.19	0.44	7.84
3.75	1115.27	0.41	10.06
4.54	1383.21	0.39	12.20
5.36	1673.23	0.37	14.40
6.24	1995.09	0.35	16.72
8.34	2812.19	0.31	22.17
12.51	4627.62	0.26	33.18
16.73	6740.79	0.22	43.85
20.9	9144.12	0.19	54.32
25.07	11,900.39	0.16	63.15
33.45	18,810.66	0.12	81.47

**Table 3:** Dimensions and shape accuracy of processed micro tubes.

Outer Diameter (mm)	Wall Thickness (mm)	Wall Thickness Uniformity (/%)	Ovality (/%)
1.00	0.07	94.0%	1.7%
1.50	0.14	95.1%	1.6%
2.00	0.12	93.0%	2.1%
2.50	0.09	94.8%	1.9%

## Data Availability

Not applicable.
